# System failure and health inequity in plain sight: Menopause, misdiagnosis, and the cost to women's physical, social, economic, mental health and wellbeing

**DOI:** 10.1016/j.cpnec.2026.100353

**Published:** 2026-05-27

**Authors:** Janet Michel, Christine Bigler, Djouroukoro Diallo, Kristina Ardojan

**Affiliations:** a Interdisziplinäres Zentrum für Geschlechterforschung (IZFG), University of Bern, Switzerland; b One Planet Sustainables, Bern, Switzerland; c Initiative Afrique, University of Bern, Switzerland; d Universitären Psychiatrischen Diensten Bern (UPD), University of Bern, Switzerland

**Keywords:** Menopause, Women's health, Misdiagnosis, Mental health impact, Call for action

## Abstract

Women make up half of the global population, yet their health continues to be overlooked, underfunded, and insufficiently studied. At the same time, they represent 70–80% of the worldwide health care workforce. As a result, Europe is not only dealing with an ageing population but also an ageing health workforce. This means the very foundation of health care systems is itself being neglected and inadequately supported. The situation is further worsened by the lack of education on menopause among health care providers, as it is often not part of the medical curriculum. This gap in knowledge, combined with limited systemic support, leaves many women in menopause feeling uncertain, overwhelmed, and alone. The consequences for both their physical and mental well-being can be significant and concerning. That is why we are calling for multi-pronged action; i) Comprehensive Menopausal Education and Training ii) Increased Ring-Fenced Research Funding iii) Women-Led Research and Empowerment iv) Menopausal-Friendly Workplace Policies and v) Allyship of All Genders.

## Highlights

• **Comprehensive Menopausal Education and Training:** Implement comprehensive menopausal education and training programs for all healthcare providers,employers, men, and women alongside widespread public education campaigns to demystify menopause and reduce associated stigma in the society.
• **Increased Ring-Fenced Research Funding:** Significantly increase and ring-fence research funding specifically for women's health in general, and menopausal health in particular. This is crucial to address the structural failures surrounding menopause and women's health globally, so as to foster evidence-based solutions.
• **Menopausal-Friendly Workplace Policies:** Develop and implement menopausal-friendly policies within workplaces to support women experiencing menopausal symptoms. This includes flexible working arrangements, access to support resources, and fostering an understanding and inclusive work environment.
• **Women-Led Research and Empowerment:** Promote and fund women-led research initiatives focused on issues that directly affect women. Simultaneously, empower women through accessible, evidence-based information and services related to their health and well-being.
• **Allyship of All Genders:** Encourage and foster the active allyship of men and all genders to support this vital cause, recognizing that gender equity in health benefits everyone.

## 1 Background

Women constitute slightly more than half of the world's population. Despite this demographic reality, menopause remains a profoundly misunderstood phase in women's lives and an under-researched topic. The global population of post-menopausal women is growing significantly; in 2021, women aged 50 and over accounted for 26% of all women and girls globally [1,2]. Projections indicate that by 2030, the number of women between ages 45 and 55 will reach nearly 500 million, meaning that roughly 6 percent of the world's population will be in menopause [3]. By 2050, the number of women aged 50 and over is expected to rise to 1.65 billion [4].

Projections indicate a need for 11-18 million Human Resources for Health (HRH) by 2030 [4]. Currently, women comprise 70-80% of the health workforce globally [5], and over half of these are aged 45 and above [6]. This demographic trend means that Europe, and indeed many other regions, faces not only an aging general population but also an ageing healthcare workforce. Furthermore, in numerous contexts, lay women, though often unremunerated, bear a significant burden of family care. While women form the backbone of our healthcare systems, very few are in leadership positions [6]. As a result, their own health, particularly menopausal health, is not adequately prioritized as a global health concern. This short communication combines elements of a narrative review, policy-oriented commentary, and an advocacy-driven position, calling for action. No systematic nor scoping review methodology was applied. The purpose of this narrative is to raise awareness, stimulate debate and garner support for women's health in general and menopause in particular.

## 2 Problem

A critical issue contributing to the neglect of women's health, including menopausal health, is the limited representation of women in leadership and decision-making positions within healthcare and policy-making bodies [6]. This underrepresentation is related to underfunding and a scarcity of research and evidence pertaining to women's health issues [7]. The resulting knowledge gaps and the absence of systemic support leave many menopausal women feeling confused, distressed, and isolated [8,9]. A central component of this problem is the widespread lack of education and training among general practitioners and gynaecologists regarding menopause. This deficiency is associated with prolonged journeys of misdiagnosis, missed diagnoses, and the prescription of inappropriate treatments, exacerbating the challenges faced by women during this life stage and their mental health in particular [2,8].

## 3 Effects

Menopause can be accompanied by substantial symptoms that can disrupt life by creating pervasive health changes [10]. The failure to recognize and adequately address menopause-related symptoms, such as anxiety, depression, cognitive fog, and emotional instability, seems to impact women across multiple dimensions: physically, socially, economically, and in terms of their mental health and overall well-being [9,11,12]. Thurston et al. posit menopause as both a biological and psychological transition associated with changes in reproductive hormones and not a disease [10]. Societal stigma and pervasive ignorance have been further reported as marginalizing menopausal women [12]. A deep-seated lack of understanding, from men, society at large, and employers regarding the complex emotional and psychological challenges women face during this transition has been associated with the perpetuation of harmful stereotypes, erosion of self-esteem, workplace discrimination, career stagnation, and even early resignations. There is an established complex relationship between mental health and well-being and menopausal symptoms such as vasomotor symptoms, sexual intimacy, cognitive function and sleep disturbances [10]. Globally, menopausal symptoms cost an estimated $150 billion in worker productivity [3,13,14]. Companies seem to be losing valuable talent, while many women report substantial financial consequences as a direct result of job losses, which in turn further worsens their mental health and well-being [14]. A study from Stanford economist, Petra Persson found that women who visit a healthcare provider with menopause-related symptoms are earning 10% less four years later or resign and leave the profession [9,15].

## 4 Call to action

**Fig. 1:**
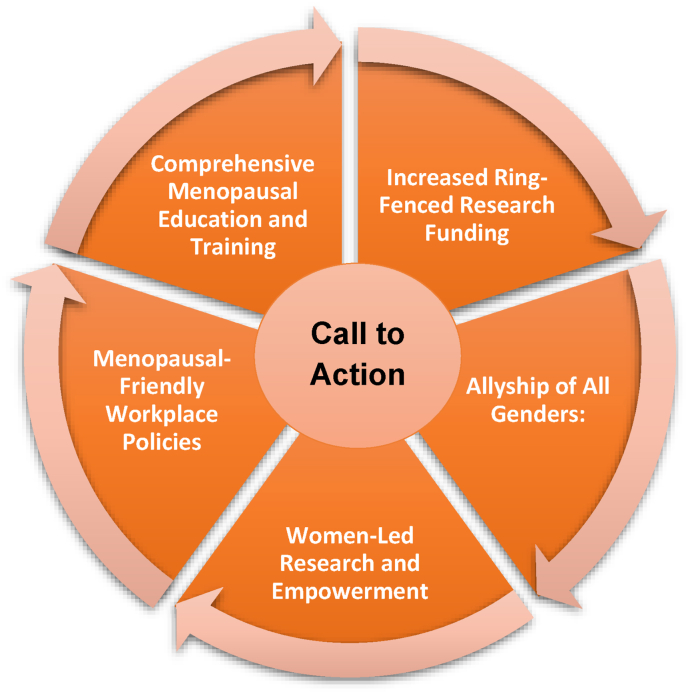
Call to action.

We must recognize and address the critical need to support our caregivers, particularly women who form the backbone of our healthcare systems and families [6]. Health care systems, health care workers, employers, and society at large have collectively failed women in this regard [6]. Therefore, we call for the following urgent actions:

I. **Comprehensive Menopausal Education and Training:** Implement comprehensive menopausal education and training programs for all health care providers, employers, men, and women alongside widespread public education campaigns to demystify menopause and reduce associated stigma in the society.
II. **Increased Ring-Fenced Research Funding:** Significantly increase and ring-fence research funding specifically for women's health in general, and menopausal health in particular. This is crucial to address the structural failures surrounding menopause and women's health globally, so as to foster evidence-based solutions.
III. **Menopausal-Friendly Workplace Policies:** Develop and implement menopausal-friendly policies within workplaces to support women experiencing menopausal symptoms. This includes flexible working arrangements, access to support resources, and fostering an understanding and inclusive work environment [16].
IV. **Women-Led Research and Empowerment:** Promote and fund women-led research initiatives focused on issues that directly affect women. Simultaneously, empower women through accessible, evidence-based information and services related to their health and well-being.
V. **Allyship of All Genders:** Encourage and foster the active allyship of men and all genders to support this vital cause, recognizing that gender equity in health benefits everyone.(see Fig. 1)

## 5 Conclusion

Menopause remains a taboo topic, under-funded and under-researched, leading women to long journeys of misdiagnosis. The impact of these misdiagnosis journeys on the mental health of women is unfathomable. Health care systems, health care workers, employers, and society at large have collectively failed women in this regard. Yet menopause can be a time of psychological growth and well-being for women [[10], [11], [12]]. Bearing in mind that women make the backbone of health care systems, the ageing population in Europe and the ageing human resources for health, action is urgently called for.

## Informed consent statement

Not applicable.

## Institutional review board statement

Not applicable.

## Data availability statement

Not applicable.

## Use of AI and AI-assisted technologies

No AI tools were utilized for this paper.

## Funding

This research received no external funding.

## CRediT authorship contribution statement

**Janet Michel:** Conceptualization, Writing – original draft, Writing – review & editing. **Christine Bigler:** Writing – review & editing. **Djouroukoro Diallo:** Writing – review & editing. **Kristina Ardojan:** Writing – review & editing.

## Declaration of competing interest

All authors declare no conflict of interest.
